# A Computational Framework for Influenza Antigenic Cartography

**DOI:** 10.1371/journal.pcbi.1000949

**Published:** 2010-10-07

**Authors:** Zhipeng Cai, Tong Zhang, Xiu-Feng Wan

**Affiliations:** 1 Department of Basic Sciences, College of Veterinary Medicine, Mississippi State University, Mississippi State, Mississippi, United States of America; 2 Department of Statistics, Rutgers University, Piscataway, New Jersey, United States of America; Imperial College London, United Kingdom

## Abstract

Influenza viruses have been responsible for large losses of lives around the world and continue to present a great public health challenge. Antigenic characterization based on hemagglutination inhibition (HI) assay is one of the routine procedures for influenza vaccine strain selection. However, HI assay is only a crude experiment reflecting the antigenic correlations among testing antigens (viruses) and reference antisera (antibodies). Moreover, antigenic characterization is usually based on more than one HI dataset. The combination of multiple datasets results in an incomplete HI matrix with many unobserved entries. This paper proposes a new computational framework for constructing an influenza antigenic cartography from this incomplete matrix, which we refer to as Matrix Completion-Multidimensional Scaling (MC-MDS). In this approach, we first reconstruct the HI matrices with viruses and antibodies using low-rank matrix completion, and then generate the two-dimensional antigenic cartography using multidimensional scaling. Moreover, for influenza HI tables with herd immunity effect (such as those from Human influenza viruses), we propose a temporal model to reduce the inherent temporal bias of HI tables caused by herd immunity. By applying our method in HI datasets containing H3N2 influenza A viruses isolated from 1968 to 2003, we identified eleven clusters of antigenic variants, representing all major antigenic drift events in these 36 years. Our results showed that both the completed HI matrix and the antigenic cartography obtained via MC-MDS are useful in identifying influenza antigenic variants and thus can be used to facilitate influenza vaccine strain selection. The webserver is available at http://sysbio.cvm.msstate.edu/AntigenMap.

## Author Summary

Influenza antigenic cartography is an analogy of geographic cartography, and it projects influenza antigens into a two- or three-dimensional map through which we can visualize and measure the antigenic distances between influenza antigens as we visualize and measure geographic distances between the cities in a geographic cartography. Thus, influenza antigenic cartography can be utilized to identify influenza antigenic variants, and it is useful for influenza vaccine strain selection. Here we develop a new computational framework for constructing influenza antigenic cartography based on hemagglutination inhibition assay, a routine antigenic characterization method in influenza surveillance and vaccine strain selection. This method can be used for antigenic characterization in vaccine strain selection for both seasonal influenza and pandemic influenza.

## Introduction

An influenza virus is a negative-stranded RNA virus that belongs to the *Orthomyxoviridae* family. There are three serotypes, A, B, and C, of which B and C are reported to infect mammals only. The influenza A viruses have 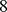 genomic segments (segment 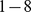) with varying lengths from about 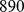 to 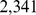 nucleotides which encode at least 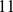 proteins: PB2 by segment 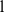, PB1 and PB1-F2 by 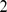, PA by 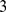, haemagglutinin (HA) by 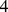, nucleoprotein (NP) by 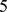, neuraminidase (NA) by 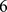, matrix protein M1 and M2 by 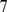, and nonstructural protein NS1 and NS2 by 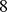. Among these proteins, the surface proteins HA and NA are involved in virus attachment and cell fusion. Both HA and NA are the primary targets for host immune systems. The serotypes of influenza A viruses are based on HA and NA subtypes. To date, 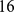 HA and 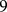 NA subtypes have been reported in influenza A viruses [1]. For instance, H1N1 influenza A virus is named since it has HA and NA recognized by HA subtype 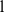 and NA subtype 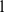 antibodies, respectively. Influenza B viruses have 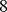 segments while Influenza C has 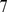 segments. There is not yet an HA-NA nomenclature system in Influenza B and C viruses.

The peak influenza season in the northern hemisphere is from January to April every year. More than 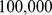 hospitalizations and 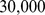 deaths are caused by influenza in the United States each year [2], [3]. The influenza A virus may cause a pandemic disaster that will impact multiple continents. In the 20th century, three influenza A pandemics occurred in 1918, 1957, and 1968, respectively [4], [5]. More than 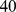 million people were killed in the 1918 influenza pandemic, which was caused by the H1N1 influenza A virus. This influenza pandemic shortened global life expectancy by more than 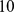 years. During March and early April 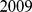, a new H1N1 influenza A virus epidemic was detected in Mexico and the United States [6], and the virus spread rapidly through human-to-human transmission, resulting in WHO declaring a pandemic, which was the first influenza pandemic in the past 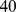 years. This virus was estimated to cause about 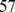 million infections and 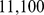 deaths solely in United States through Jan 14, 2010 (www.cdc.gov). If we consider all cases in five continents, the numbers will become significantly larger.

In the United States, vaccination is the primary option for reducing the effects of influenza. The seasonal influenza vaccines used in the past decades include three viral components: H1N1 influenza A virus, H3N2 influenza A virus, and influenza B virus. In an effective vaccination program, vaccine strain selection will be the most important step since the highest protection could be achieved only if there is an identical antigenic match of the vaccine and epidemic virus HA and NA antigens, especially HA, which is the primary target of human immune system. However, as an RNA virus, influenza A virus has rapid mutations in these two proteins, and such mutations can cause a change of antigenicity, thus making vaccines ineffective. Mutations in HA and NA are also referred as antigenic drift.

Immunological tests, such as hemagglutination inhibition (HI) assay, enzyme-linked immunosorbent assay (ELISA), and microneutralization assay, have been utilized to identify antigenic variants among the circulating influenza strains. Among these assays, HI, has been one of the routine procedures in influenza vaccine strain selection. HI assay is an experiment to measure how a testing influenza antigen (virus) and a reference antiserum (antibody) react. The antibody is usually diluted in 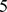 fold first and then diluted in powers of 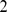. Thus, the titre from HI assay will be 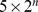, 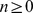. The larger the 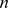 is, the more closely the testing antigens match the reference antigens, for which the reference antisera are generated. Usually a number smaller than 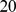 is considered as a low reaction between antigen and antibody. In many cases, HI experiments are used to measure the antigenic distance between two testing antigens through their immunological reactions to the same reference antiserum. For instance, if one testing antigen is a high reactor for the reference antiserum (e.g. with a titre of 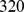) while another testing antigen is a low reactor (e.g. with a titre of 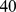). The antigenic distance could be approximately 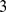 units, which is 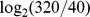. In reality, the antigenic distances are usually measured by a set of reference antisera, thus the calculation is much more complicated. Such measurements from HI data are generally used to determine the antigenic distances between testing antigens.

**Figure 1:**
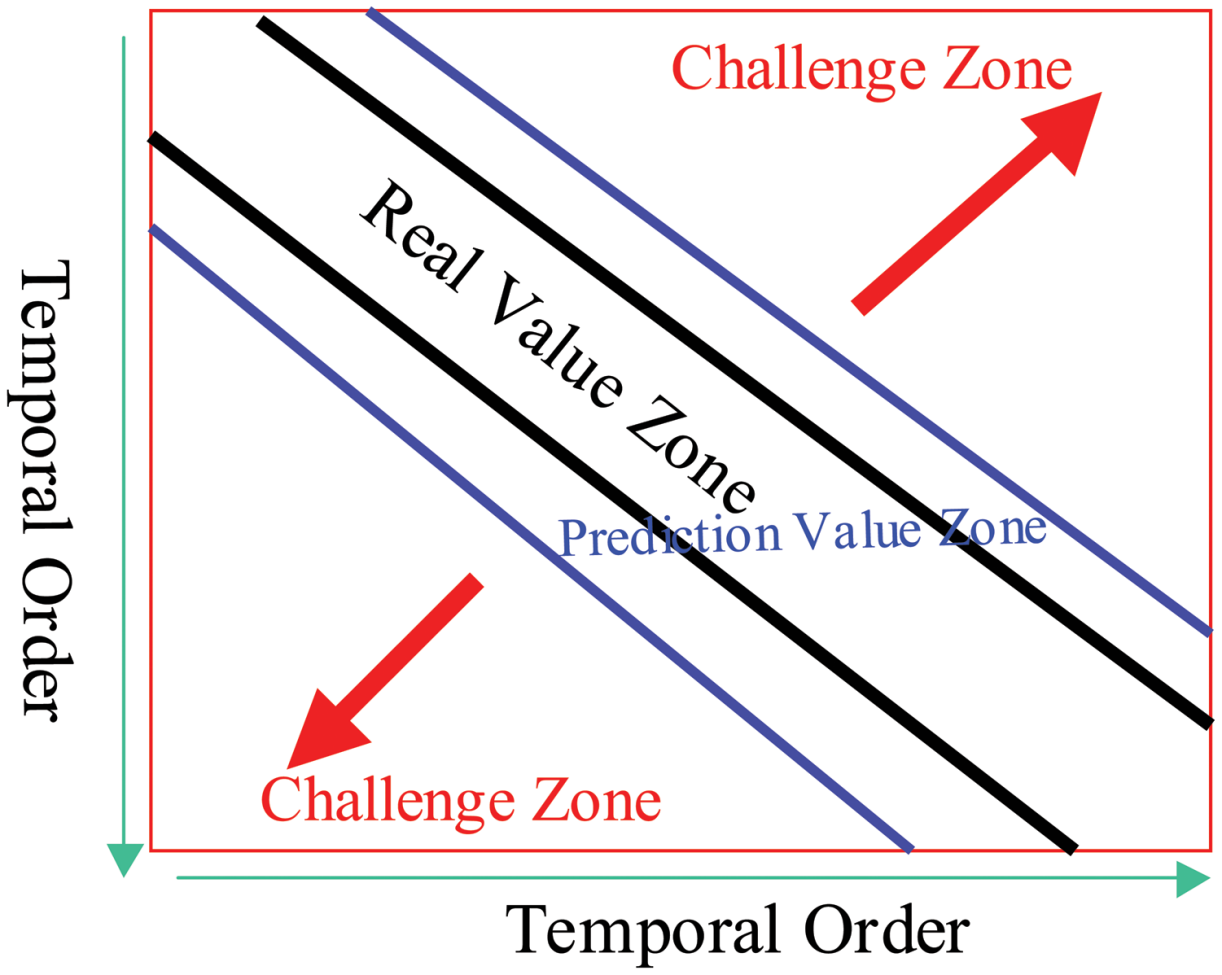
The hemagglutination inhibition (HI) data in temporal order. High reactor are in the diagonal zone, and the low reactors and the missing values will appear more when the approaching the challenge zone.

In a typical influenza HI assay, generally less than 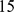 reference antisera are used but the number of test antigens can be more than 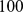. However, interpretations of HI results are not straightforward due to the following two challenges: (1) HI assay only shows the indirect relationship between antigens and antisera since each value reflects a reaction from antigen, red blood cell (RBC), and antibody. Many variables from RBC and antibody will interfere the HI titres; (2) it is not be possible to perform HI for all pairs of antigen and antisera reactions. Thus, the resulting HI table is generally incomplete, and the percentage of missing data could be up to 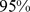. By applying the metric multidimensional scaling method (MDS) to reduce the shape space into less than three dimensions, Lapedes and Farber [7] showed a linear correlation between logarithm values of HI titers and the space distances between influenza antigens. Based on this method, Smith *et al.* [8] constructed influenza cartography to visualize the distances among influenza antigens from HI tables by further developing the metric MDS method. Their method assumes that antigens and antibodies are mapped into the same low-dimensional space, and their interactions are the distances between the embedded points. However, in our implementation of their algorithm, the resulting influenza cartography depends on the initial values selected, and thus may not be stable. Moreover, this method results in cartographies in which global distances may contain relatively large errors. This is because the algorithm does not incorporate temporal modeling to reduce the inherent temporal bias in HI tables. The temporal bias is caused by the fact that HI table entries are not missing uniformly at random, and off diagonal entries are more likely to be missing or become low reactors (Figure 1). The underlying biological reason for this bias can be explained by the herd immunity effect, where influenza antigens evolve rapidly under the accumulating immune pressures of human population [9]. A more detailed illustration of this phenomenon will be given later.

The goal of this paper is to present a computational framework for influenza cartography construction which we call Matrix Completion-Multidimensional Scaling (MC-MDS). An important aspect of this framework is that temporal modeling can be easily incorporated, which as we shall show, is useful for dealing with HI tables with herd immunity induced temporal bias. Our framework includes two integrated steps: (1) a low rank matrix completion algorithm is first employed to fill in the entries of the HI matrix; (2) a MDS algorithm is utilized to map the antigens (or similarly, antibodies) into a two dimensional space for visualization. Our approach explicitly separates the visualization (cartography) step from the matrix completion step, making it easier to incorporate temporal models. Our experience shows that while temporal modeling is beneficial in both steps, it is less important in the first step, for which we may simply employ a sliding window approach; however it is more essential in the second step, for which we propose a more complex herd-immunity temporal regularization model as described in the Materials and Methods section. The reason for the difference is that the inherent temporal bias tends to give rise to incorrect global distances if not handled explicitly, and thus affect the 2D cartography process more significantly. The two step procedure in our approach is thus flexible in the first step, where we can simply use a standard low rank matrix completion algorithm. On the other hand, we have to pay special attention to temporal modeling in the second step, which is essential for accurate cartography construction. Both simulation and a practical application in H3N2 influenza A viruses demonstrate that this method is able to overcome some limitations in the original metric MDS method of [8] and it results in better influenza antigenic cartographies from HI data. Therefore the proposed framework can potentially facilitate more accurate interpretation of HI data in influenza surveillance as well as more accurate identification of influenza antigenic variants. Both are essential for influenza vaccine strain selection.

## Results

While greater details are given in the Materials and Methods section, we shall summarize the most important observations and intuitions in our computational framework before presenting the actual experimental results.

### Characteristics of HI data

In this work we are specifically interested in HI datasets existing accumulating original, such as the immunological datasets of human origin. In a typical HI dataset, three types of data entries are present: Type I, a regular HI titre; Type II (low reactors), the value is defined as “less than a threshold”, e.g. 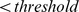 and this threshold is caused by the lower bound experimental limit in HI assays indicating a weak (or low) immunological reaction between a testing antigen (virus) and an antiserum (antibody); Type III, missing values. A major characteristic of HI dataset is that the distributions of type I, type II, and type III data are not random. Specifically, if we arrange both antigens and antibodies in a HI matrix according to time, then there is a banded structure, where most Type I data appear very close to the diagonal of the matrix; Type II data tend to be slightly off diagonal, while Type III data are more likely to occur in matrix entries that are significantly off diagonal (Figure 1). This data characteristic introduces a “temporal bias” concerning the data distribution (in comparison to uniformly random distribution) that needs to be corrected. As we will show, if the problem is not handled appropriately, then inaccurate result will be produced. This is because classical methods assume uniformly random data distribution, which does not take the temporal bias effect into consideration. Our paper shows that temporal modeling, which reduces the data distribution bias in HI tables, is important in HI based influenza cartography.

**Figure 2:**
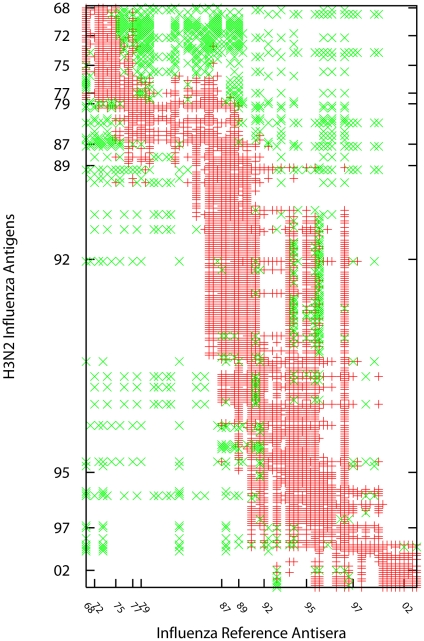
Data distribution in the H3N2 HI dataset. Three types of data are present in HI data: type I, a regular HI titre marked in red cross; Type II, the value is defined as ‘less than a threshold’, e.g. 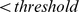, where 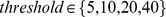, and these values indicate the testing antigen and antiserum have a weak (or low) immunological reaction. Type II values are marked in green diagonal cross; Type III, missing values, which are blank. This HI dataset includes 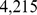 entries, which represent 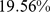 data presence. Among these entries, 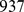 (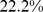) are Type II values.

The specific benchmark dataset used in our study includes 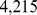 entries, representing 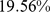 of all table entries (Figure 2). Among these entries, 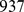 (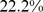) are Type II values (that is, they are recorded as 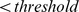) with 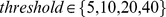. For algorithmic comparison purposes, we also include results on a simulation dataset with ground truth, which is generated according to characteristics of real HI datasets.

### Sliding-window procedure for matrix completion

As pointed out above, most Type I data are located across the diagonal line of the HI matrix, which significantly deviates from the “missing uniformly at random” assumption in classical matrix completion. In order to reduce this bias, we adopt a sliding window approach where each low rank matrix completion will be performed in a HI sub-matrix, which has fewer amount of Type II and Type III data that more closely satisfy the “missing uniformly at random” assumption. The remaining entries that are not covered by the (sliding window) sub-matrices can be filled with a global matrix completion algorithm – those entries will be predicted with less accuracy due to the banded-structure of the HI data that violates the “missing uniformly at random” assumption.

The windows are based on the temporal spans of influenza A viruses. In order to complete the entire matrix, the algorithm will slide yearly along with both the dimensions of antigens and antisera to ensure the time difference between all antigens and antisera are within a certain window size. In order to obtain an optimal window size and best rank in matrix competition, we tested six different sizes, including 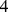, 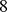, 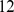, 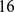, 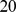, and 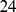, and ranks 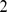 to 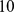. A 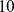-fold cross validation suggested that the time frames of 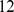-year and 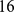-year with rank 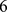 are two best ones towards achieving the lowest RMSE (root mean squared error) value in matrix completion of H3N2 dataset (Table 1). The average RMSE from 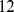-year experiment is slightly better than that from 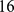-year experiment. Both the average RMSE for 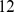-year and 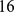-year experiment are better than that from the entire HI matrix. Thus, during matrix completion, a window of 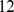 and a rank of 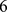 will used. Similarly, our optimization method demonstrated that the window size of 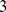 and the rank of 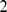 are the best parameters for our simulation data.

**Table 1:** The local RMSE values from 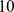-fold cross validations using H3N2 HI dataset (1968–2003) with different window sizes (W) in sliding window based Alternating Gradient Descent.

	Rank								
W	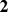	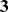	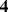	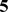	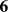	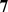	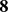	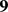	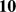
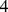	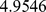	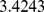	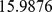	–	–	–	–	–	–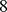	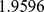	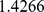	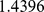	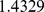	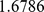	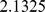	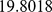	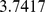	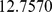
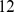	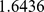	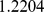	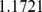	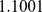	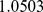	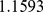	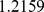	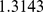	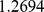
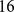	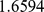	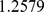	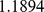	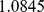	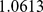	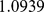	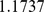	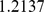	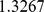
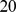	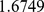	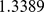	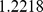	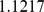	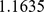	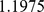	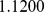	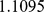	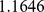
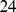	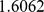	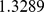	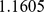	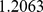	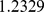	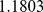	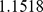	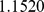	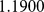

### Herd-immunity MDS model for antigenic cartography construction

After the matrix completion step, we need to project the influenza antigens onto a two-dimensional (2D) map. In order to obtain accurate global distances, we incorporate a temporal model in MDS based on the fact that the influenza antigens continue to evolve under the accumulating immune pressures of human population [9]. In order to evade the herd immunity, an influenza virus will most likely evolve into a strain with different antigenicity from recently circulating strains in human population. This intuition is mathematically incorporated in our temporal MDS model, where we assume that on the 2D cartography, influenza viruses tend to evolve along (approximate) straight-line segments during short time spans; that is, they tend to evolve in directions as far away from recently appeared viruses as possible. The detailed mathematical formula is presented in the Materials and Methods section.

In HI tables, a Type II value is resulted from experimental limitation of HI assay and reflects a weak (or low) immunological reaction between a testing antigen/antiserum pair. Although this value is not as informative as a Type I value, it is more useful than a Type III value (missing value). In particular, if a particular virus has type I values with a certain set of antibodies that show strong reactions, while another virus reacts weakly with the same set of antibodies (resulting in type II values), then the global distance between their 2D cartography embeddings should be relatively large. A set of constraints on global distances can be derived from this observation. The details can be found in the Materials and Methods section.

There are four parameters 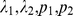 to be optimized in our temporal MDS model. We use 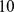-fold cross validations to select the optimal parameters that achieve the lowest RMSE while satisfying global distance constraints derived from Type II data. Our cross-validation results led to 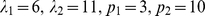 for the real data and 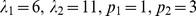 for the simulation data.

### Influenza cartography construction using simulation data

To demonstrate the potential impacts of Type II data (low reactors) and Type III data (missing values) on the influenza cartography, we performed experiments using simulated HI matrices containing 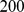 antigens versus 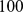 antibodies in which we know the ground-truth. Three simulated HI matrices were generated, where one was based on the distributions of H3N2 1968–2003 HI data: (1) HI matrix (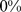 data absence) with neither Type II nor Type III data; (2) HI matrix (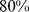 data absence, data structure: randomly distributed) with Type III data but without Type II data; (3) HI matrix (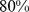 data absence, data structure: with a temporal data missing bias similar to H3N2 data as shown in Figure 2) with both Type II data and Type III data. The first HI matrix serves as the benchmark data (ground truth). The second HI matrix is used to test the efficiency of standard matrix completion algorithms under the missing uniformly at random assumption. The third matrix is used to examine the efficacy of the temporal model in MDS. A more effective computational method would be expected to produce a cartography more similar to that of the benchmark matrix. Using these simulated HI matrices, we are able to compare the MC-MDS method proposed in this work to the original metric MDS method of [8] in terms of HI matrix completion and cartography construction accuracies.

To assess whether MC-MDS and metric MDS can accurately recover the HI values in the HI data, we calculated the local RMSEs for the Type I data using 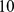-fold cross validation (Table 2). The experimental data were partitioned into 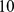 parts, and each time we use 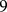 parts for training and 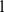 part for testing. The RMSE values were calculated using the Type I values in the testing part. Here we only use Type I values for RMSE calculation in order to be consistent with our real-data experiment, where we do not know the ground-truth corresponding to Type II and Type III data. The local RMSE values were 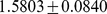 for MC-MDS and 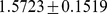 for metric MDS, where the notation of 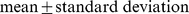 is used. Since a typical matrix value is about 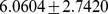, these local RMSE values indicate that both methods were able to recover HI values effectively. The small difference between the two means of MC-MDS and metric MDS is significantly smaller than the standard deviations. Hence they are statistically insignificant. However, we note that metric MDS has a larger standard deviation, which is consistent with our observation that it is less stable.

**Table 2:** Comparison between MC-MDS and metric MDS.

		HI recovering	Cartography construction		
		Local RMSE^1^	Robustness	Global distance measurement	
			Correlation coefficient (CC value)^2^	Maximum distance(MD value)^3^	Pairwise distance RMSE (PD value)^4^
Simulation	MC-MDS	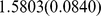 5	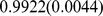	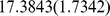	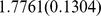
	Metric MDS	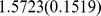	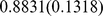	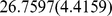	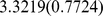
H3N2	MC-MDS	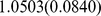	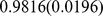	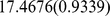	—
	Metric MDS	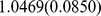	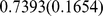	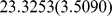	—

1 HI recovery ability is assessed by calculating the RMSE values on the Type I data using 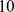-fold cross validation, and these values are also called local RMSEs.

2 A correlation coefficient (CC value) is calculated from the pairwise distances among antigens for every two independent runs. The CC values in this table were calculated from 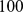 different runs.

3 A maximum distance (MD value) refers to the difference between the maximum distance among any antigens in the benchmark cartography and that from the method being evaluated (either MC-MDS or metric MDS). The MD values in this table were calculated from 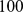 different runs.

4 A pairwise distance RMSE (PD value) is the difference between the pairwise distances among all antigens in the benchmark cartography and those from the method being evaluated. The PD values in this table were calculated from 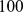 different runs. The PD values for H3N2 data were not assessed since we do not know the ground truth of antigenic cartography for this dataset.

5 The value in the bracket is the standard deviation of the associated parameter.

The effectiveness of a cartography construction algorithm can be assessed using figures of merit that measure its robustness and correctness. The robustness of a method is determined by the correlation coefficient (CC value) that is calculated from the pairwise distances among antigens for every two independent runs. The correctness of cartography is measured by two values: the difference between the maximum distances (MD value) between any antigens in the benchmark cartography and that from the method being evaluated (either MC-MDS or metric MDS); the pairwise distance RMSEs (PD value), calculated by measuring the difference between the pairwise distances among all antigens in the benchmark cartography and those from the method being evaluated. We performed 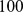 independent runs, and the mean and standard deviation for each figure of merit can be found in Table 2.

**Figure 3:**
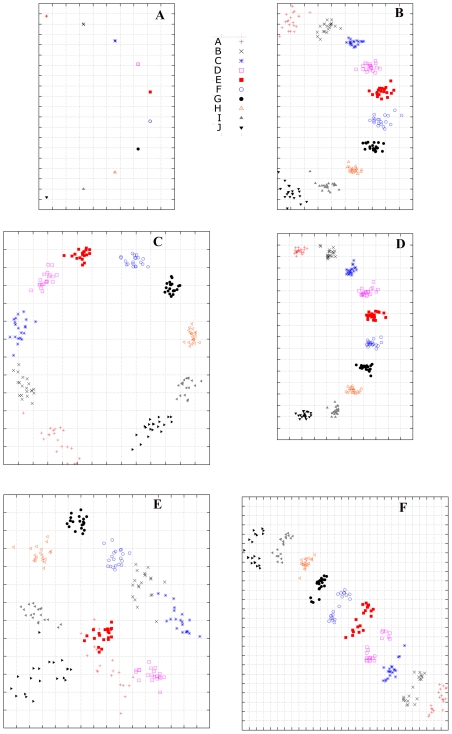
Computational simulation demonstrates that temporal model can reduce the biases generated by the Type II data (low reactors) in hemagglutination inhibition (HI) dataset. (a) HI matrix (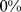 data absense) with neither Type II nor Type III data, using multidimensional scaling (MDS); (b) HI matrix (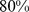 data absense, data structure: randomly distributed) with Type III data but without Type II data, using Alternating Gradient Descent (AGD) and MDS; (c) HI matrix (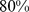 data absense, data structure: similar to H3N2 data as shown in Figure 1) with both Type II data and Type III data, using AGD and MDS; (d)HI matrix (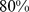 data absense, data structure: similar to H3N2 data as shown in Figure 1) with both Type II and Type III data, using MC-MDS. (e)HI matrix (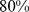 data absense, data structure: similar to H3N2 data as shown in Figure 1) with both Type II and Type III data, using Metric MDS. (f) Another independent run by the same setting and method as (e).

As specified in the Materials and Methods section, the matrix completion method employed in this paper was Alternating Gradient Descent (AGD). In Figure 3, the ground-truth cartography is given in Figure 3a. Figure 3b shows a typical result when matrix entries are missing uniformly at random (the second matrix generated in our simulation study), where the standard AGD method accurately reconstructed cartography since the resulting cartography is similar to that from the benchmark matrix. Figure 3c shows a typical result with temporally biased HI table (the third matrix generated in our simulation study), where the cartography was constructed from a combination of AGD for matrix completion and the conventional MDS (without temporal modeling) for cartography generation. It shows that this combination is unable to accurately recover the cartography of the benchmark data since the global distances are incorrect. In comparison, the combination of AGD with temporal MDS, shown in Figure 3d, does achieve significantly more accurate global cartography. This experiment demonstrates the need to explicitly incorporate temporal modeling into the MDS step. Moreover, our experiment shows that cartographies generated by AGD and temporal MDS are stable. The CC value and PD value for the 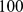 independent runs are 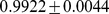 and 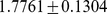, respectively. The MD value for the 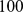 independent runs is 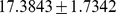, which is close to the ground-truth value of 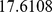 in the benchmark cartography (Figure 3a).

For comparison, we implemented the metric MDS method of [8] and applied to the third HI matrix which was generated with temporally biased data type distributions. Our results indicate that the cartographies from metric MDS are less stable, with two typical runs given in Figure 3e and 3f. In the 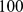 independent runs of metric MDS, the CC value and PD value for the 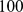 independent runs are 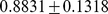 and 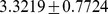. The MD value is 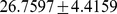. These numbers are significantly worse than the corresponding numbers from the MC-MDS method proposed in this work. We shall especially note that the metric MDS method tends to over-estimate the global distances in this stimulation study. Moreover, the large standard deviations in the results also indicate that metric MDS is not very stable.

While these two methods achieve similar matrix completion accuracies, the reconstructed cartographies reveal a more significant difference. As we pointed out earlier, this is because the temporal bias (of data type distribution) in HI tables has stronger impact in the MDS step, especially when we compare global distances. Without temporal modeling, the accuracy of global distances between two points (representing two viruses) in the 2D cartography decays more rapidly when the two points become further apart in time. While this reduction of accuracy is an unavoidable limitation of the banded structure in HI tables (Figure 1) that makes it harder to reliably compare points far away in time, a good temporal model can alleviate its impact, and thus increase the accuracy of the resulting cartography.

Finally we summarize the main observations from this simulation study as follows. Both MC-MDS and metric MDS methods achieved similar accuracy in recovering HI values. This means that they achieve comparable performance in the matrix completion sub-task, which is less sensitive to the temporal bias problem in HI tables. However, without temporal modeling, the global distances among far away points in the reconstructed cartography become inaccurate. Therefore it is helpful to incorporate temporal modeling into the MDS step in order to reduce the temporal bias effect. The proposed MC-MDS framework (with herd-immunity temporal model) is effective in reducing the bias problem, and it leads to more accurate cartography. The metric MDS appears to be less stable and it generates less accurate cartographies because the method does not address the temporal bias problem.

### Influenza antigenic cartography for H3N2 influenza A virus

**Figure 4:**
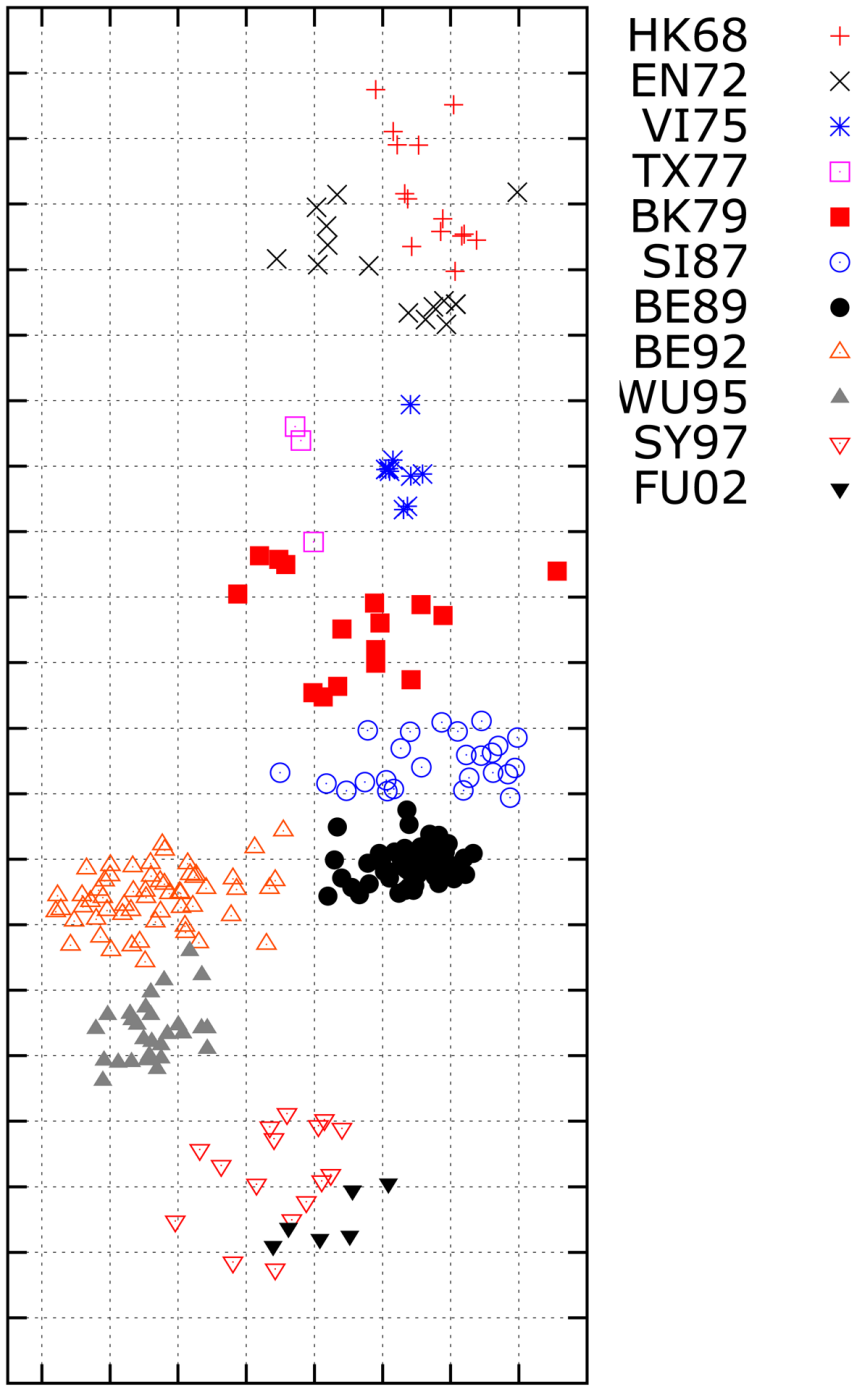
The influenza antigenic cartography constructed by MC-MDS for H3N2 viruses from 1968 to 2003. The 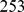 viruses labeled with the cluster names HK68, EN72, VI75, TX77, BK79, SI87, BE89, BE92, WU95, SY97 and FU02 are defined by [8]. One unit (grid) corresponds to a two-fold change in HI assay.

In the second experiment, we use MC-MDS to construct influenza cartography for H3N2 influenza A viruses from 1968 to 2003 using the HI datasets from Smith *et al.* [8]. The antigenic map is shown in Figure 4. The scale of antigenic cartography is based on the antigenic distances from HI tables, e.g. each unit (grid) in the antigenic cartography represents of a 2-fold change in HI titres. These viruses are specifically labeled as eleven clusters (HK68, EN72, VI75, TX77, BK79, SI87, BE89, and BE92, WU95, SY97, and FU02). Our results indicate that the antigenic distance between HK68 and FU02 is approximately 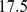 units.

**Figure 5:**
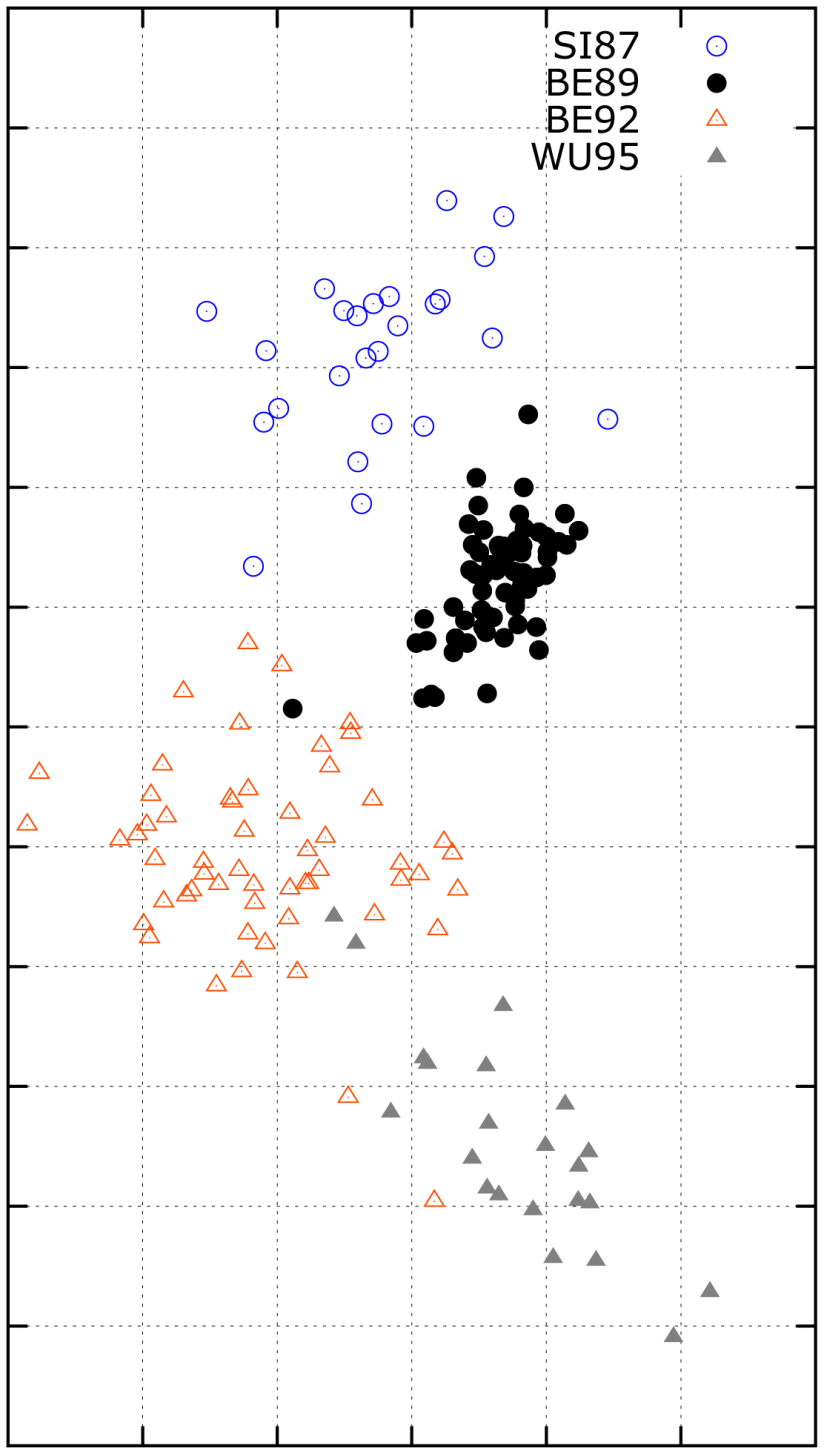
The antigenic cartography by MC-MDS for H3N2 HI data from 1987 to 1995. The influenza viruses labeled with the cluster names SI87, BE89, BE92, WU95 are defined by [8]. One unit (grid) corresponds to a two-fold change in HI assay. This data is a subset of the HI data shown in Figure 2.

The resulting cartography can be compared to the published antigenic map in Smith *et al.* [8]. The overall trend in our results is similar to the cartography from Smith *et al.* [8]. However, there are two major differences: (1) The global distances in our cartography are smaller than those of Smith *et al.* [8]. For example Smith *et al.* [8] shows a distance of 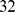 units between HK68 and FU02. Although we have no ground truth for this data, we note that this discrepancy is consistent with our simulation study, where the metric MDS method also produces larger global distances. In that case, the metric MDS method over-estimated the global antigenic distance between A and J by 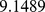 units more than the true distance. (2) The local cartographies between some clusters are different. For instance, the distance between WU95 and BE89 from our method is larger than those shown in Smith *et al.* [8]. In order to examine which antigenic cartography is likely to be more accurate, we performed a small cartography for H3N2 HI data from 1987 to 1995. Since the number of Type II data on the HI data from 1987 to 1995 is quite small, the effects of Type II on the antigenic cartography is minimal. Therefore, the cartography for the viruses between 1987 to 1995 using data from the limited span will not suffer much from the temporal bias problem discussed in the paper, and thus should be close to the true cartography. Our result shows that the distance between WU95 and BE89 should indeed be larger than that between BE95 and BE92 (Figure 5), and this is consistent with the local cartographies from MC-MDS.

Similar to the simulated HI data experiments, we can assess the robustness of MC-MDS and metric MDS on the H3N2 data (Table 2). The best local RMSE was 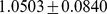 for MC-MDS and 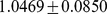 for metric MDS. Therefore there is no statistically significant difference in matrix completion quality. The CC values from the 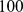 independent runs are 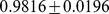 and 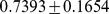 for MC-MDS and metric MDS, respectively. The MD value was 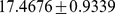 for MC-MDS and 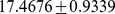 for metric MDS. These numbers are consistent with the simulation study, showing again that MC-MDS is more stable for antigenic cartography construction.

From the 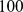 runs of metric MDS, we were not able to generate the exact cartography in Smith et al. [8]. One reason might be that the initial values we randomly chose were not exactly the same as those from [8], which were not specified clearly from [8]. The source code of our implementation of the metric MDS method in [8] is available upon request. We shall point out that our implementation is strictly based on what was described in [8]. While we have spent great effort to ensure the correctness of our implementation, it is possible that there are undocumented improvements in the optimization algorithm used to solve the metric-MDS problem. In such case, their actual implementation might not suffer from the issues observed in our study. Nevertheless it is still useful for us to examine problems of the algorithm presented in their original paper, the underlying causes of these problems and their potential mathematical remedies. This is what this study tries to achieve.

## Discussions

Each year, about 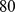 World Health Organization (WHO) collaborating laboratories and 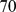 National Respiratory and Enteric Virus Surveillance System (NREVSS) that are located throughout the United States participate in virologic surveillance for influenza. By collaborating with over 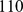 other National Influenza Centers in the WHO Global Influenza Surveillance Network, the vaccine strains for next influenza season are determined in the middle of February each year for northern hemisphere (these strains are used as vaccine strains in the United States) and September for southern hemisphere. The pandemic vaccine strains are also selected through collaborative efforts among different laboratories across the WHO Global Influenza Surveillance Network. Influenza vaccine strain selection is a very labor intensive procedure that depends on both antigenic characterization and genetic characterization. In general, whether an isolate will be sequenced or not is based on the result from antigenic characterization, and only highly potential antigenic variants are sequenced. Therefore, antigenic characterization is critical for vaccine strain selection. In order to identify a potential influenza vaccine strain, we have to integrate the HI tables from different experiments in the same laboratories or even from different laboratories. Each experiment only includes up to 15 reference antisera, which are updated at each influenza season or even each month within the same influenza season. In addition, it is common for individual laboratories to use different antisera. Therefore, the integrated HI table is typically an incomplete matrix. This incompleteness and the limitation of HI experiments (see the introduction section) present a challenge in interpreting HI results and thus antigenic variant identification. Another important challenge of HI data is the temporal bias effect, which means that entries in an HI matrix are not missing uniformly at random (Figure 1). These are the problems this paper addresses.

As an analog of geographic cartography, influenza cartography can be used to visualize and measure antigenic distances between influenza viruses. An essential criterion for a new influenza vaccine strain is significant antigenic divergence (e.g. 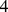 fold change in HI test) from the current vaccine strain. Influenza antigenic cartography can help us identify whether a testing antigen (virus) is antigenically far away from a specific vaccine strain or a specific cluster of antigens (e.g. circulating strains at a specific time period).

In this study, we proposed a new computational framework for constructing an influenza antigenic cartography, and demonstrated its usefulness in antigenic characterization. This computational framework has two integrated steps: (1) through a matrix completion algorithm, influenza antigenic distance matrices are constructed; (2) through MDS (with herd-immunity temporal model), influenza antigens (viruses) are projected onto a two-dimensional cartography. We specifically pay attention to the major challenge that is caused by the temporal bias in HI datasets. That is, the banded structure of HI entries indicates that the matrix entries are not missing uniformly at random (Figure 1), which violates the standard assumption in conventional methods. Our experiment showed that standard approach will not handle this problem very well, and will produce cartographies with incorrect global distances. This paper addresses the problem through a biologically motivated temporal evolution model that is mathematically incorporated into the MDS algorithm. It is shown that more accurate antigenic distances can be obtained from this approach.

Although MC-MDS is presented as a 2D cartography construction method in this paper, it can be extended easily for 3D (or even higher dimensional) cartography by modifying the resulting cartography dimension in the MDS step of our computational framework.

The temporal regularization in MC-MDS is based on the fact that the influenza antigens continue to evolve under the accumulating immune pressures of human population [9]. Within a short time period, the antigenic distances among viruses tend to become larger in temporal order. Such a regularization is important since it can effectively minimize the biases of Type II data. However, such regularization does not necessarily imply that the antigen would always evolve forward. Theoretically, it is possible that the antigenicity (not genetic sequence) of influenza viruses could become similar to earlier circulating strains when the selective pressure from herd immunity disappears. This is supported indirectly by the report that 2009 pandemic H1N1 virus cross-reacted with the serum from the ages over 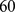, who were likely to infect the seasonal H1N1 virus circulating in human population before 1957 [10].

Besides the immunological datasets for the influenza viruses (such as those of human origin) with the accumulating immunity from their hosts, there are other immunological datasets for the influenza viruses from mutations (not necessarily accumulating immunity), such as those of swine or avian origin. For the latter case (e.g. the data of swine or avian origin), our limited experiments in H5 and H7 studies suggested that the users can use MC-MDS directly without temporal model (data not shown). However, there might be additional structures to explore in such data. This requires more extensive investigations in the future.

### Conclusion

We introduced a new computational framework for influenza antigenic cartography construction from HI datasets. This approach, which we refer to as MC-MDS, integrates two mathematical procedures: matrix completion and MDS projection (with temporal modeling). Using the AGD matrix completion algorithm on HI datasets from 1968 to 2003, we successfully identified the eleven reported clusters of antigenic variants that represent major antigenic drift events during these 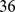 years. Thus, this method is useful in both influenza antigenic variant identification and influenza vaccine strain selection. Our results also demonstrated that MC-MDS is more robust and effective than our implementation of the metric MDS method [8] in influenza antigenic cartography construction.

## Materials and Methods

### Dataset and data transformation

#### H3N2 HI benchmark dataset and data transformation

The benchmark HI dataset is adopted from [8], and it includes 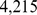 observed HI values from the reactions from 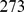 H3N2 influenza A viruses against 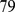 ferret antisera. These viruses were isolated periodically from locations around the world between 1968 and 2003, and the antisera were generated against 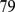 prototype influenza strains, most of which were selected from these 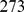 influenza isolates. Both influenza antigen (virus) and antiserum (antibody) can be roughly clustered into eleven groups, HK68, EN72, VI75, TX77, BK79, SI87, BE89, BE92, WU95, SY97 and FU02, which represent the eleven major events of antigenic drifts resulting in a pandemic or an epidemic from 1968 to 2003. For instance, FU02 represents a group of influenza viruses isolated around the year of 2002 with similar antigenic characteristics.

Within this dataset, three types of data points are present: Type I, a regular HI titre; Type II, the value is defined as ‘less than a threshold’, e.g. 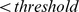, where 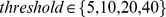, and this value represents the testing antigen and antiserum do not strongly react with each other; Type III, missing values. Following [8], we preprocess the HI matrix by normalizing the data entries as follows: each Type I entry with the observed value 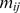 is transformed to 
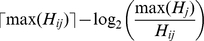
, where 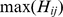 is the largest HI value among all observed entries and 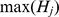 is the maximum HI value for antiserum 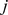; each Type II entry with value 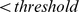 is transformed into 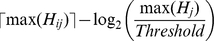; Type III data are replaced with 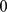 s, representing the missing values.

#### Simulated HI data

To study the effect of temporal bias on influenza cartography, we simulate HI matrices with 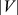 viruses and 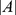 antibodies. Both the viruses and antibodies can be partitioned into 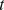 blocks by temporal information. In each block, we will have 20 viruses and 10 antibodies. Let 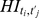 denote the HI titre for 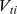 and 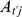, where 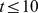, 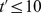, 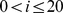, and 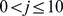. Each 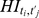 is a random value generated uniformly from 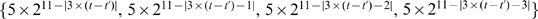.

In the HI matrix with Type II values, all the HI values no more than a titre of 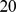 will be replaced with Type II values of the form 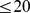. The matrix is preprocessed according to the same method used in the H3N2 HI benchmark dataset. To generate incomplete HI matrices, we randomly select Type I and Type II HI values (about 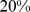) from the entire HI matrix by mimicking the data distribution in the H3N2 HI dataset.

## Matrix completion algorithms

The goal of matrix completion is to fill the missing entries in an incomplete matrix based on appropriate mathematical models of the matrix. It is a traditional mathematical problem that has been studied for many decades. Early contributions on this problem include Schur [11], Farahat and Ledermann [12], Friedland [13], Hershkowitz [14], London and Minc [15], Mirsky [16] and Oliveira [17]–[19]. In the past decade, interest in the problem has grown substantially, especially after the launch of Netflix competition [20] in 2007. The Netflix problem is to predict each user's movie preference (in order for Netflix to make appropriate movie recommendations to each user) from approximately 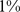 observed user ratings. This can be regarded as a matrix completion problem, where we predict missing user/movie ratings from incomplete observations. This is exactly like the problem of predicting antigen/antibody interactions which we consider in this paper. In general, matrix completion is ill-posed and computationally intractable [21], [22]. However, recently, Candes and Rect [22] and Recht *et. al* [23] proved that under appropriate conditions, the minimum rank matrix solution can be recovered from incomplete entries by solving a convex optimization problem. These theoretical developments generated further interest, and afterwards, a number of new methods have been proposed [24]–[30].

If we do not consider the temporal bias effect, then the antigenic cartography task can be formulated as a matrix completion problem. Simply, in an HI matrix, there are 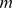 antigens corresponding to the rows, and 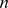 antisera corresponding to the columns. Let 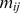 denotes the HI value from the reaction between testing antigen 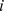 and antiserum 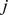. The HI matrix can be represented as
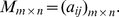
Let 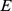 denote the subset of 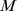
's entries corresponding to Type I and Type II data. In practice 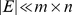. The goal of matrix completion is to estimate the HI values in the Type II and Type III entries as accurately as possible. In addition, matrix completion can re-estimate Type I entries and remove embedded noises, which were from the uncertainties in experimental measurements. This is a standard matrix completion problem. The standard approach to this problem is to assume that the matrix is low rank, with rank 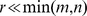. In our application, this means that each antigen 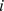 can be embedded into the 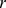-dimensional space as 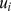, and each antiserum 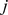 can be embedded into the 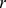-dimensional space 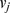. In the low rank model, the interaction 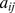 between antigen 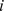 and antiserum 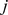 is given by 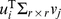 for some matrices 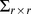. We can aggregate vectors 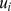 into a matrix 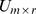 and aggregate vectors 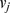 into a matrix 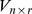. Mathematically, the low-rank model is to find matrices 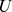 with dimensions 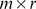, 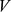 with dimensions 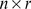 and a diagonal matrix 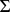 with dimensions 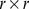 such that
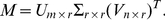
(1)


Here we describe AGD matrix completion method, which is developed based on gradient decent method. AGD method assumes the low rank matrix completion model (1).

If type II data are not present, one can employ the following optimization formulation to estimate the missing values
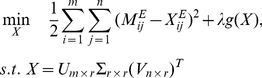
(2)where 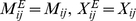 when 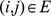 and 
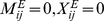
 otherwise.

The function 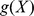 is a regularization condition for the matrix 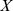, which is introduced to stabilize the solution. The solution 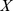 of the optimization problem (2), which does not contain any missing value, will replace 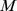 (which has missing values) as the true (and denoised) HI table, which we can then use for other purposes, such as cartography construction.

In the AGD method, we take 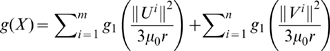 in (2), where 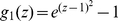 when 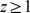 and 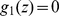 otherwise. 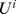 (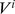) denotes the 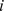
th row of 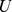
(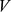) and 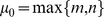.

First, the algorithm uses SVD to obtain the factorization 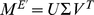. Here 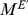 is the trimmed matrix of 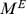 where we randomly set some observed values to 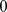 from the rows (columns) when a row (column) contains more than 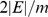
(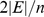) observed values. The purpose of this trimming step is to guarantee that each row (column) has less than 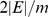
(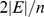) non zero values. This is based on the observations of Keshavan *et. al.* [27] that when 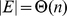, the corresponding singular vectors are highly concentrated on high-weight column (or row) indices. It means that those vectors do not provide useful information. After SVD, we set the initial value 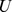 to 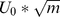 and 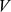 to 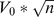 where 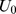 and 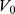 are the first 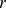 columns of 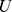 and 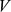 respectively.

We then apply the following alternating optimization procedure until convergence or when certain number of iterations are reached.

- Fix 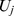 and 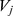 and calculate the matrix 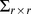 to minimize the squared error 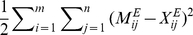. This is a least squares regression problem with respect to 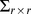.
- Update 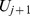 (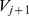) using gradient descent: we take steps proportional to its negative of gradient with respect to the objective function. That is, 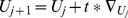 and 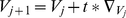 where 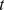 is the step size parameter which can be optimized by line search algorithm.
- The first two steps are repeated until convergence or reaching a pre-defined number of iterations.

The gradient of 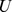 and 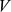 are:
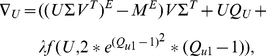
(3)

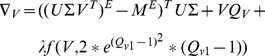
(4)where
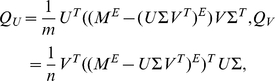


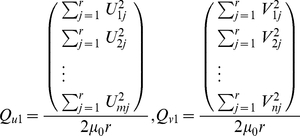
and
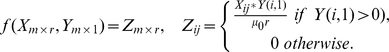



### 

#### A general method to handle Type II values

Although Type II data is not as informative as Type I data, they still provide useful information. Therefore we have to modify (2) to include type II data. First we introduce threshold values 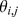 for each entries 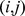, and let 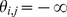 if the corresponding entry is not type II data. If an entry is type II data, we set 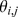 to be the corresponding threshold. We change the standard matrix completion formulation (2) into the following form that incorporates type II information:
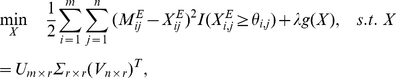
(5)where 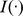 is the indicator function: 
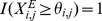
 if 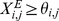; and 
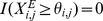
 if 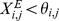. The intuition behind this formulation is that for an type II entry 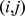, if 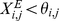, then we do not have to penalize the error 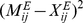 because the constraint is satisfied.

One advantage of this formulation is that we can employ any optimization algorithm that solves (2) to solve (5). We start with an initial estimate of 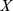 by ignoring the type II data. We then iterate as follows until a certain number of iterations are reached:

- Let 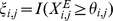

- Update 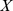 by solving
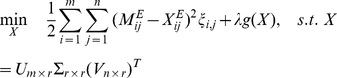
using any optimization algorithm (such as AGD) for (2).

The procedure is a principled approach to handle Type II data, and it can be used with any algorithm that optimizes (2).

The two parameters, 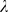 in the penalty function and the rank to project data, are trained through 10-fold cross validation. The rank (from 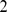 to 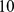) and 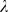 (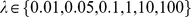) with the smallest RMSE value will be selected as the best rank in matrix completion.

#### Performance evaluation

The performance of matrix completion is evaluated using the following three criteria in this study: root mean squared error (RMSE), correlation coefficient, and biological interpretation. Given 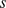 values 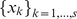 and 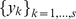, we define the RMSE as:
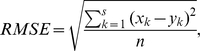
where 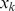 stands for an observed value and 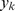 stands for the corresponding predicted value. If a prediction scheme has a small RMSE value, then the predicted values are close to the true values. For matrix completion, we utilize 10-fold cross validation to calculate the RMSE values. The observed matrix entries are partitioned into 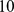 equal parts. Each time, one part is used for testing and the other nine parts for training. That is, each time we use 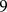 parts as observed values in matrix completion; after the completed matrix is generated from these 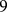 parts, we calculate the RMSE between the completed matrix and the observed matrix entries in the remaining part. The process is repeated for every part in the dataset. The RMSE value is the average RMSE value over several runs. The RMSE values were estimated only using Type I values, and thus they are also called local RMSE. Note that we also report the standard deviation numbers calculated from cross validation. It is known that standard deviation calculation based on cross validation is often smaller than the true standard deviation; however, the numbers still provide meaningful indications and hence are included.

For the temporal based MDS, we can define the distance between two viruses as the Euclidean norm between the rows of the completed HI table corresponding to the two viruses. In evaluation, we use the local pairwise distances among temporally close by viruses because these distances are more reliable. In particular, the local pairwise distances are partitioned into 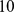 equal parts. Again, each time we left one part as testing samples and applied temporal based MDS by using 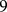 parts. The RMSE between the estimated distance and testing data are calculated, and they are called the pairwise distance RMSEs (PD value).

The correlation coefficient (CC) between two vectors measures the strength and direction of their linear relationship. Let 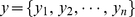 denote the vector A and 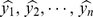 denote the vector B. The correlation coefficient (CC) between vector A and B is defined as follows:
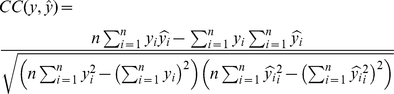



Clearly, a larger CC value indicates the two vectors are closely related. For every two runs, we will have two distance vectors and one CC value. In 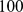 experiments, we will have 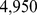 CC values, then the mean and standard deviation can be calculated. This test is to assess whether every two runs of the test method (temporal MC-MDS or Metric MDS) are different. For instance, if metric MDS has lower CC value and larger standard deviation, we will conclude it is not stable.

The biological interpretation is based on separation and quantification of the reported antigenic variant groups in the influenza antigenic cartography.

#### Window size determination

In order to reduce the temporal bias in HI matrices, we adopt a sliding window approach in the matrix completion step. The rational for sliding window matrix completion is that the temporal bias effect becomes much smaller in temporally grouped sub-matrices than in the entire HI matrix. This means that the effect of temporal bias will be reduced when we complete each sub-matrix separately. Therefore in our approach low rank matrix completion will be performed separately in each HI sub-matrix. In order to complete the entire matrix, the algorithm will slide yearly along with both the dimensions of antigen and antisera to ensure the time difference between all antigen and antisera are within a certain window size 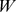. The missing values or low reactors in HI matrix are estimated as the mean value of the recovering values from the associated sub-matrices. If the missing values or low reactors are not covered by any of the sub-matrices, they will be estimated through matrix completion using the entire HI matrix. The selection of window size 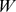 is based on minimizing the RMSE values from 10-fold cross validation. For H3N2 HI dataset, we have tested different values of 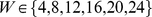.

#### Temporal based MDS for Type II data

Multidimensional scaling (MDS) is a statistical technique widely used in information visualization. It embeds a set of data into low dimension vectors while preserving their pair-wise distances. The projection of viruses into two or three dimensional space can be viewed as an analog of a geographic cartography; thus this is referred to as influenza antigenic cartography. Due to the temporal bias effect in HI tables, we have to incorporate a temporal model into the MDS algorithm to reconstruct global distances more accurately. In this work, we consider a biologically motivated temporal regularization criterion. The regulation in our temporal model is based on the fact that the influenza antigens continue to evolve under the accumulating immune pressures of human population [9]. In order to evade the herd immunity, an influenza virus will most likely evolve into a strain with different antigenicity from recently circulating strains in human population. Thus, within a certain time period (e.g. 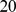 years, which is within one human generation), the antigenic distances among viruses tend to become larger in temporal order.

This intuition is mathematically incorporated into our temporal regularization condition. Specifically we assume that on the 2D cartography, influenza viruses tend to evolve along (approximate) straight-line segments during short time spans; that is, they tend to evolve in directions as far away from recently appeared viruses as possible. The concrete mathematical formulation is described below.

First we denote by 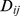 the average distance between virus 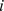 and virus 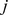 using the completed matrix from step 1, and let 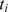 denote the isolation year of virus 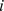. We partition all influenza viruses into 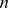 groups based on their temporal ordering. If a dataset covers year 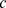 to year 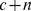, then the 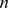 groups are viruses in year 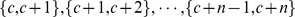. This grouping choice is due to the fact that each influenza season in the northern hemisphere spans two years. Therefore without additional information, it is appropriate to assign every virus to the neighboring years as well. For instance, it is natural to assume that the A/Beijing/32/92(H3N2) virus belongs both to group 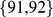, and to group 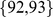. Denote by 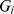 the viruses in the 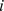-th group. In 2D cartography, we represent each virus by a two dimensional vector. Let 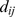 be the distance between virus 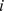 and virus 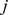 in the cartography; let 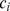 be the center coordinate of group 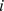 and hence 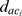 is the distance between virus 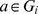 to the center 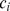, and 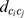 is the distance between two centers 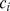 and 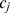. The temporal MDS method in our experiments attempts to minimize the following error function:
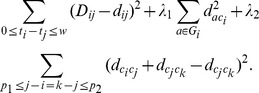



The first term is the standard MDS. The second term means that viruses within each group should be close to each other. The third term is the mathematical formulation that formalizes the biological intuition that viruses tend to evolve along straight-line segments during short time spans.

Besides the above error function, we impose constraints on global distances that can be derived from the original dataset. We know that each reference antiserum is associated to an antigen. Let the reference antiserum be 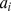 and its corresponding antigen be 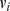. If antigen 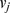 has low reactor (Type II) with this reference antiserum 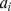 and antigen 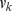 has relatively high reactor with 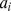, we can naturally assume that in the 2D cartography, the distance between 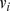 and 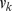 should be smaller than the distance between 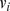 and 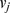. In our experiments, if a HI value is equal to or more than 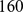 (4-fold higher than the Type II threshold 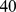), we call it a relatively high reactor. The four parameters 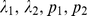 are optimized using 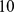-fold cross validations. The configuration that achieves the best RMSE for reconstructing local pairwise distances while satisfying all constraints is selected. The source codes for this implementation are available upon request.

#### Metric MDS for influenza antigenic cartography construction

The metric MDS method is developed by Smith *et. al.* [8] to construct antigenic cartography. This method attempts to minimize an error function of the form 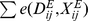. Here 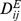 is set to 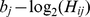 where 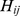 is the observed value and 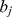 is the 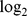 of the maximum reaction for antiserum 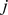. The error function is defined as
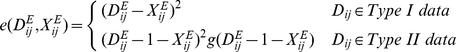
where 
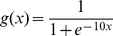
.

Similar to AGD , this algorithm also requires a pre-defined dimension (rank) 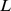 as input; that is, each virus or antiserum is represented by an 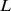-dimensional vector. Let 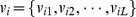 and 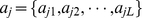 represent virus 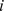 and antiserum 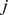, respectively. The 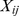 is defined as the Euclidean distance between the vector 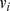 and 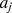: 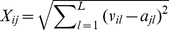. The algorithm generates the random initial vector for each virus or antiserum and the solution is found through conjugate gradient optimization with multiple random restarts.
